# Colon perforation as a complication of COVID-19: a case report

**DOI:** 10.1186/s40792-021-01261-0

**Published:** 2021-08-04

**Authors:** Keita Nakatsutsumi, Akira Endo, Hiraaki Okuzawa, Iichiro Onishi, Anri Koyanagi, Eiki Nagaoka, Koji Morishita, Junichi Aiboshi, Yasuhiro Otomo

**Affiliations:** 1 Trauma and Acute Critical Care Center, Tokyo Medical and Dental University Hospital of Medicine, 1-5-45 Yushima, Bunkyo-ku, Tokyo, 113-8510 Japan; 2 Department of Acute Critical Care and Disaster Medicine, Graduate School of Medical and Dental Sciences, Tokyo Medical and Dental University, 1-5-45 Yushima, Bunkyo-ku, Tokyo, 113-8510 Japan; 3 Department of Diagnostic Pathology, Tokyo Medical and Dental University Hospital of Medicine, 1-5-45 Yushima, Bunkyo-ku, Tokyo, 113-8510 Japan; 4 Department of Comprehensive Pathology, Tokyo Medical and Dental University Hospital of Medicine, 1-5-45 Yushima, Bunkyo-ku, Tokyo, 113-8510 Japan; 5 Department of Cardiovascular Surgery, Tokyo Medical and Dental University Hospital of Medicine, 1-5-45 Yushima, Bunkyo-ku, Tokyo, 113-8510 Japan

**Keywords:** COVID-19, Intensive care, Coagulopathy, Intestinal perforation, Intestinal ischemia, Emergency surgery

## Abstract

**Background:**

Coagulopathy induced by COVID-19 has received much attention. Arterial and venous thrombosis of multiple organs due to COVID-19-related coagulopathy is associated with a poor outcome.

**Case presentation:**

A 67-year-female was transferred to our hospital in need of intensive care for severe COVID-19 pneumonia. On day 7 after admission, despite the treatments, her respiratory and hemodynamic status deteriorated. Computed tomography revealed massive ascites and free air as well as wall defects of the transverse colon. An emergency laparotomy was undertaken in the intensive-care unit, and 17 cm of the transverse colon was resected. Histopathological findings revealed two perforation sites of 25 and 7 mm in diameter, necrosis of the intestinal mucosa around the perforation sites, and the microcirculatory thrombosis in the mesentery vessels which was suspected of having been induced by COVID-19-related coagulopathy.

**Conclusions:**

The case highlights the risk of intestinal ischemia and perforation induced by COVID-19 coagulopathy. Physicians treating COVID-19 should recognize the risk and evaluate patients carefully.

## Background

Several cases of pneumonia caused by an unknown pathogen were reported from China in December 2019. Later, severe acute respiratory syndrome coronavirus 2 was identified as the causative pathogen, and the diseases caused by this virus was defined as coronavirus disease 2019 (COVID-19) [1, 2]. COVID-19 has rapidly spread around the world and remains an unprecedented global health problem.

Coagulopathy induced by COVID-19 has received much attention. This coagulopathy can cause arterial and venous thrombosis of multiple organs including the lung, limb, brain, and intestine, which result a poor patient outcome [3, 4].

We herein report a patient who underwent emergency surgery for transverse colon perforation due to intestinal ischemia induced by COIVD-19-related coagulopathy.

## Case presentation

A 67-year-old female with a 4-day history of a fever and cough was admitted to a local hospital for pneumonia. The diagnosis of COVID-19 was confirmed by a positive polymerase chain reaction (PCR) test result via a nasopharyngeal swab. Her respiratory condition gradually worsened after admission. Thus, she underwent intubation and was transferred to our hospital in need of intensive care 5 days after admission.

**Fig. 1:**
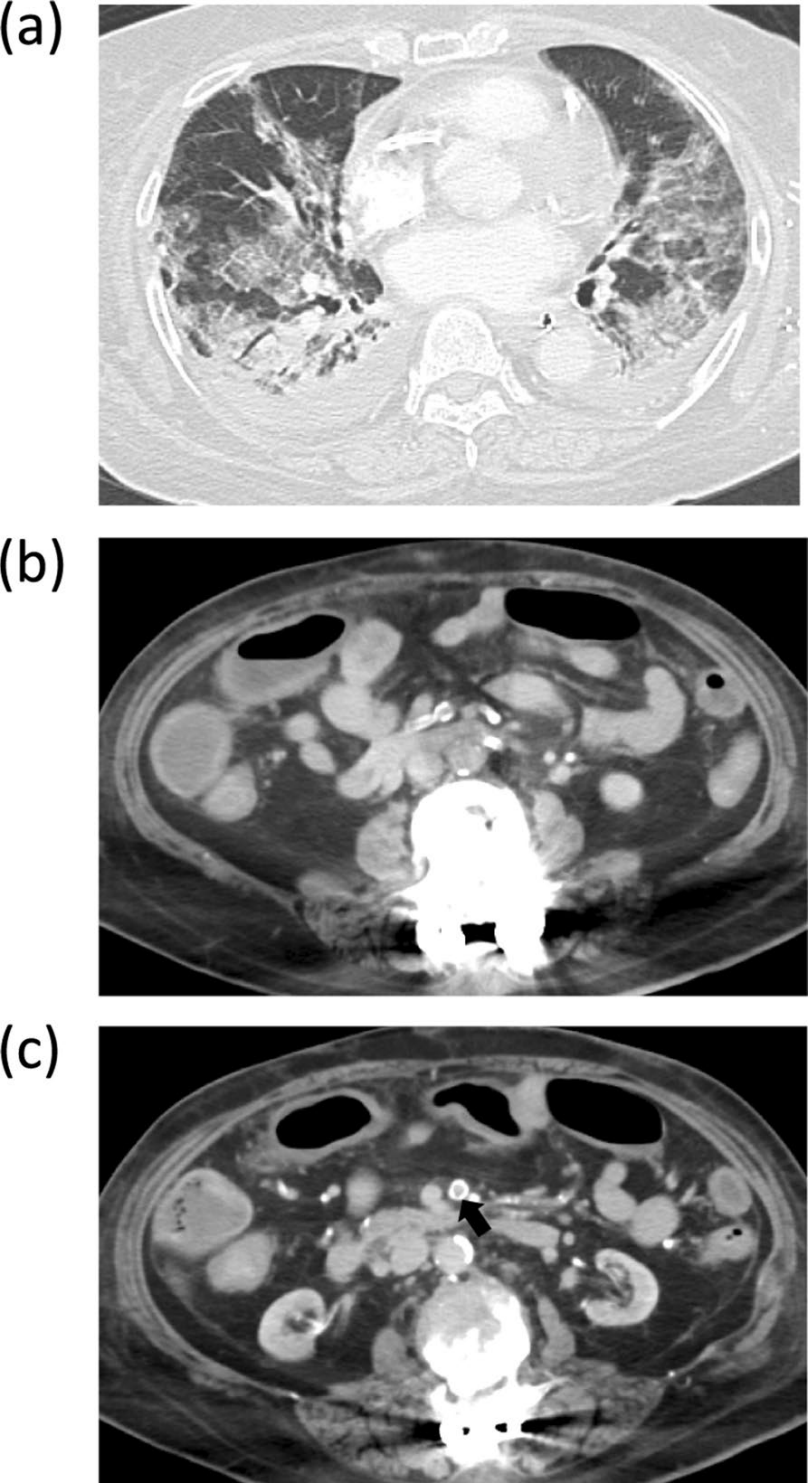
Computed tomography on admission showed bilateral ground-glass opacity with lower-lung predominance (**a**). There were no obvious abnormal findings in the abdominal region or thromboembolism. The intestine, including the transverse colon was edematous (**b**). The superior mesenteric artery showed strong sclerotic changes (**c**, arrow)

Her height and weight were 164.9 cm and 46 kg, respectively. She had comorbidities of diabetes mellitus, diabetic nephropathy requiring dialysis, angina, post-resection gastric cancer and postoperative spinal canal stenosis. She was taking several regular medications including an antiplatelet agent. On arrival, a physical examination revealed a body temperature (BT) of 34 °C, blood pressure (BP) of 110/65 mmHg, heart rate (HR) of 102 beats/min, and Glasgow Coma Scale of E1VtM1 with deep sedation. The arterial blood gas analysis showed pH 7.309, partial pressure of carbon dioxide (PaCO_2_) 41.5 mmHg, partial pressure of oxygen (PaO_2_) 78.2 mmHg with pressure control mechanical ventilation set as follows: positive end-expiratory pressure (PEEP) of 8 cmH_2_O, peak inspiratory pressure (PiP) of 25 cmH_2_O, fraction of inspired O_2_ (FiO_2_) of 0.5. The laboratory results were as follows: white blood cell counts (WBC) of 13,900/μl, C-reactive protein (CRP) of 13.1 mg/dL, D-dimmer of 7.25 µg/mL, activated partial thromboplastin time (APTT) of 170 s, and international normalized ratio (INR) of 1.35. Computed tomography (CT) revealed bilateral ground-glass opacity with lower-lung predominance. There were no evident abnormal findings in the abdominal region or thromboembolism; however, the intestine, including the transverse colon was edematous, and the abdominal vessels showed strong sclerotic changes (Fig. 1). Dexamethasone administration (6 mg/day), started at the previous hospital, was continued. Continuous renal replacement therapy was initiated for the chronic renal failure as well as appropriate body fluid management. On day 3 after admission, antibiotic therapy by cefepime was started for ventilator-associated pneumonia. The APTT decreased to 51.5 s, thus unfractionated heparin for prophylactic-dose anticoagulation was additionally administered to keep the APTT around 60 s.

**Fig. 2:**
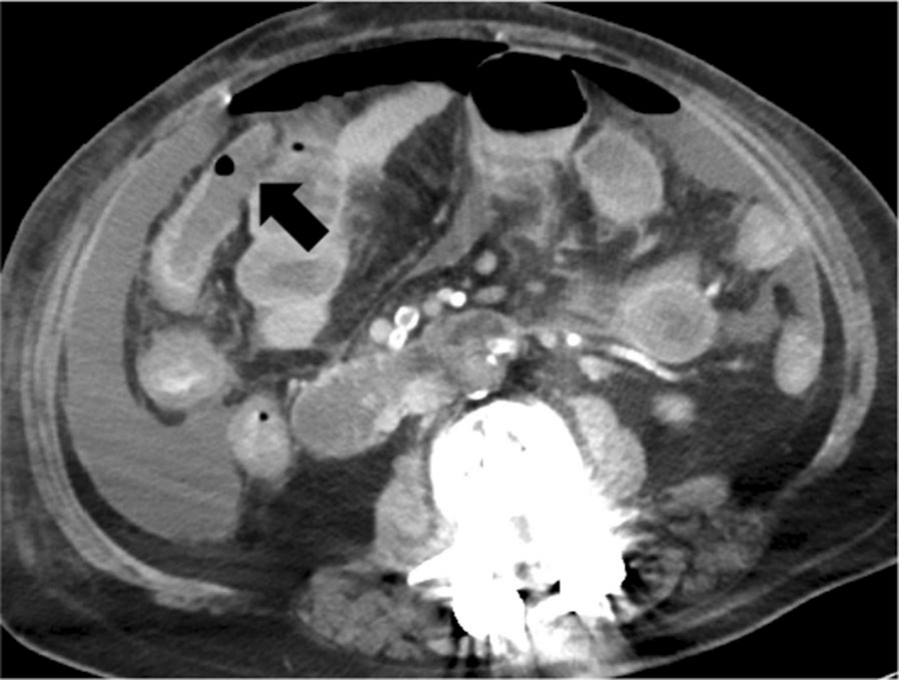
Computed tomography before emergency surgery showed massive ascites and free air as well as wall defects of the transverse colon (arrow)

On day 7 after admission, despite these treatments, her respiratory condition worsened as follows: pH 7.333, PaCO_2_ 40.6 mmHg, PaO_2_ 71.1 mmHg under the ventilator setting of PEEP of 10 cmH_2_O, PiP of 22 cmH_2_O, FiO_2_ of 0.8. Furthermore, hemodynamic deterioration also developed with a BP of 85/41 mmHg and HR of 108 beats/min under the noradrenaline administration (0.2 µg/kg/min). Laboratory tests revealed an increase in the inflammatory markers and derangements in the coagulative function as follows: WBC of 15,100 /µl, CRP of 32.14 mg/dL, D-dimmer of 26.51 µg/mL, APTT of 47.2 s, and PT-INR of 1.24. Therefore, follow-up CT was performed to re-evaluate the degree of lung injury and to detect other sources of infection. CT revealed massive ascites, free air, and wall defects of the transverse colon (Fig. 2). Emergency laparotomy as the source control of pan-peritonitis due to intestinal perforation was performed with the extracorporeal membrane oxygenation (ECMO) team on standby, as her respiratory condition was close to the limit of being able to be supported by a ventilator only. All surgical procedures were undertaken in the negative-pressure room of the intensive-care unit (ICU), considering the risks related to patient transfer such as further deterioration of the patient’s condition and pathogen exposure to the medical staff.

**Fig. 3:**
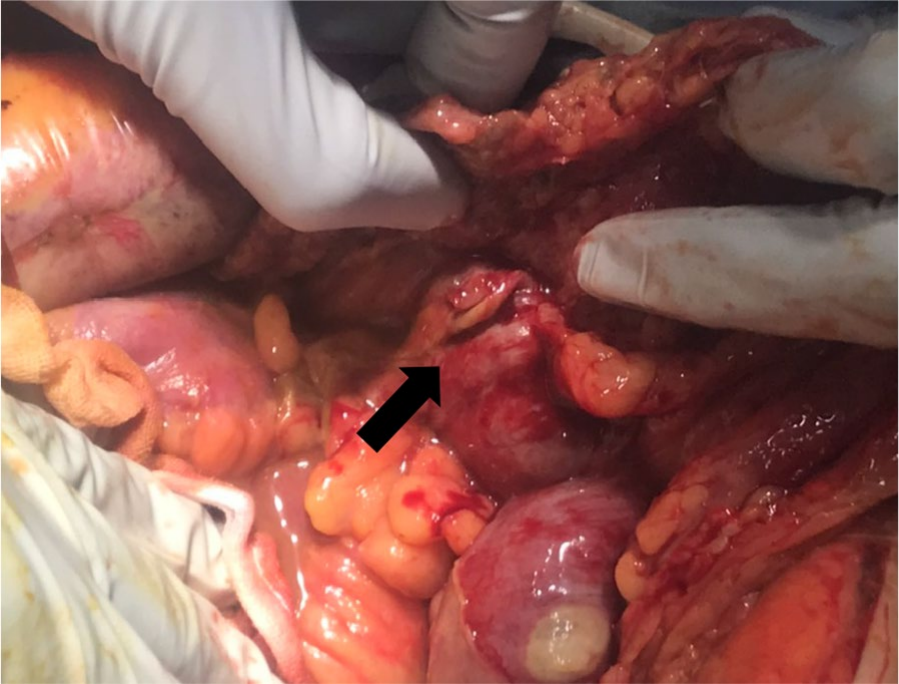
During an operation, perforation sites were identified at the right side of the transverse colon (arrow) and the tissue around the perforation sites was necrotic

**Fig. 4:**
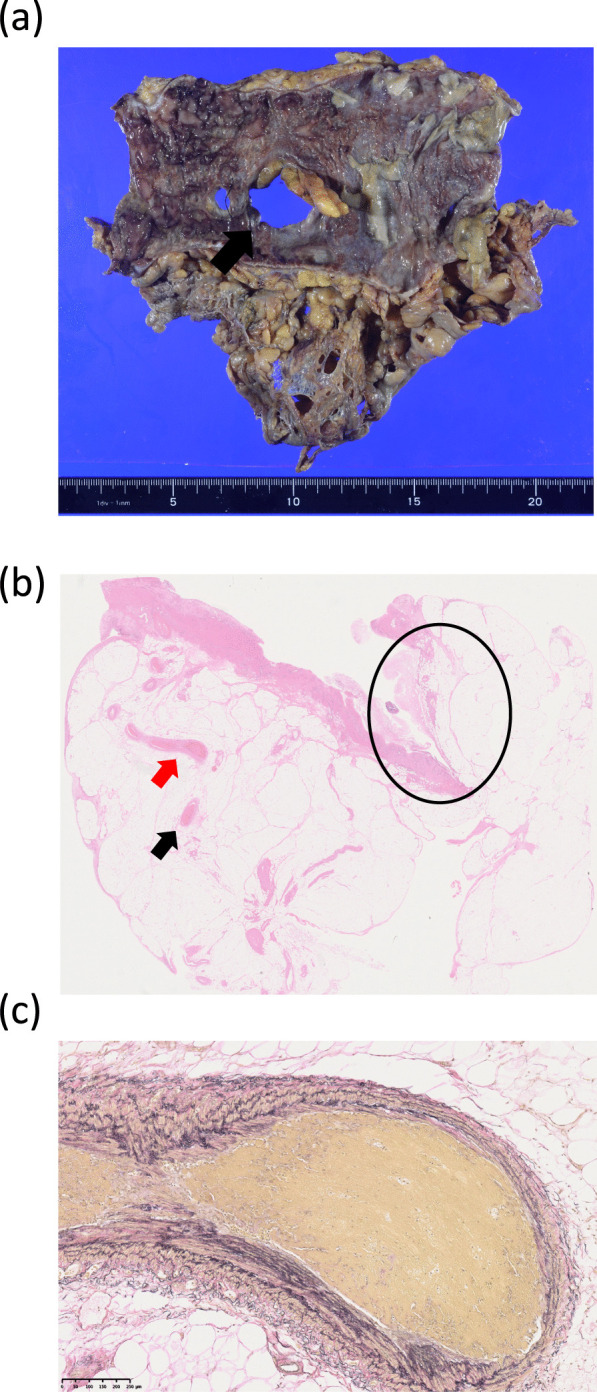
Pathological findings of the resected specimen. A total of 17 cm of the transverse colon was resected, and 2 perforation sites of 25 and 7 mm in diameter were identified (arrow). The mucosa around the perforation sites was necrotic (**a**). The area enclosed in the circle, shows the perforation site. Microcirculatory thrombosis was found in the mesenteric veins (arrow) (**b**, HE staining, ×3.9). Higher-power field of microcirculatory thrombosis in the mesenteric vein, indicated by a red arrow in b (**c**, EVG staining, ×77)

A midline skin incision was performed, and the abdomen was filled with contaminated ascites. Two perforation sites of 25 mm and 7 mm in diameter were identified at the right side of the transverse colon, and the tissue around the perforation sites changed necrotic (Fig. 3). PCR for the ascites showed a positive result and the number of copies of the virus was 42,056 (the number of copies of the virus in the sputum: 501,420). Abdominal lavage and partial resection of 17 cm of the transverse colon were performed. Considering the hemodynamic instability of the patient, open abdominal management with ABTHERA™ (KCI, now part of 3 M Company, San Antonio, TX, USA) and a planned relaparotomy strategy was selected. The secondary surgery was performed 2 days after the first operation. The abdomen was uncontaminated, and no remnant ischemic lesion was observed. Thus, colostomy was done, and the abdominal incision was closed with several drainage tubes into the abdomen. A histopathological examination revealed necrosis of the intestinal mucosa around the perforation sites and microcirculatory thrombosis in the mesentery veins, which was suspected of having been induced by COVID-19-related coagulopathy (Fig. 4).

Enteral feeding was re-started on postoperative day 2. All drains were removed on postoperative day 7. Abdominal complications, such as surgical site infection, remnant abscess and stump leakage, were not noted; however, the COVID-19 pneumonia ultimately progressed, and she died due of respiratory failure 24 days after admission (17 days after the initial surgery).

## Discussion

We reported a rare case of colon perforation as a complication of COVID-19 pneumonia. Previously reported cases of intestinal ischemia or perforation induced by COVID-19 coagulopathy have been scarce [5–7]. The exact prevalence of ischemia or perforation induced by COVID-19 coagulopathy is unclear. Bchayana et al. examined abdominal imaging findings of patients with COVID-19. A total of 134 patients were evaluated. In 13 cases, intestinal ischemic changes were detected, and only 1 case was diagnosed with intestinal perforation [8].

The mechanisms that cause coagulopathy related to COVID-19 have not been fully elucidated. Consumptive coagulopathy, a typical feature of disseminated intravascular coagulation, is usually not seen in COVID-19. The direct infection of endothelial cells by coronavirus through angiotensin-converting enzyme 2 and the high concentration of proinflammatory cytokines, such as tumor necrosis factor-α and interleukins, activated by COVID-19 have been considered to play important roles in COVID-19-related coagulopathy [9, 10]. Furthermore, the results of coagulation tests in cases of COVID-19-related coagulopathy demonstrate distinctive characteristics. While the levels of D-dimmer markedly increase, the decrease in the platelet count is relatively modest, leading to the presence of a hypercoagulable state and the increased risk of thromboembolism [10]. A previous study showed that thrombotic events occurred in 16% of hospitalized COVID-19 patients, of whom 6.2% were venous and 11.1% arterial. The mortality of patients with thrombotic events was higher than that of the whole patients (43% vs. 21%) [11]. The indication of antithrombotic therapies for COVID-19 has been still unclear. While prophylactic-dose anticoagulation for COVID-19 patients is recommended in a guideline [12], there is insufficient evidence regarding the effect of therapeutic-dose anticoagulation for COVID-19 patients without any sign of thromboembolism [13]. In the present case, although the prophylactic-dose of unfractionated heparin was used, the thrombotic event failed to be prevented.

The cause of colonic perforation varies, including colonic pseudo-obstruction, diverticulitis, stercoral perforation and medication-induced perforation. In COVID-19 cases, colonic perforation caused by interleukin-6 receptor antagonist therapy was also reported [14]. However, in the present case, these causes were unlikely based on the radiological and surgical findings. The histopathological findings of the present case were similar to those of previously reported cases of COVID-19-induced intestinal ischemia or perforation: partial or total necrosis of the intestinal mucosa and vascular change, such as congestion and organized thrombi [5–7]. These ischemic changes differed from the findings observed in the intestinal ischemia caused by critically ill conditions, such as nonocclusive mesenteric ischemia. We therefore concluded that COVID-19-related coagulopathy in addition to the patient’s poor vascular condition due to diabetes mellitus had caused intestinal ischemia and perforation in the present case.

Since surgery for patients with COVID-19 expose staff to a high risk of cross-infection, preoperative planning to minimize the risk is very important [15]. A previous COVID-19 patient underwent emergency surgery in an ICU to avoid unnecessary spread of the virus that might occur by transferring the patient to the operating room, as with the present case [16], which might be an option for reducing the risk of viral transmission. Surgical procedures also carry concerns of infecting the operation staff. Indeed, the PCR test of the ascites collected during surgery showed many copies of the virus in the present cases, which was consistent with a previous study reporting that the feces of the COVID-19 patients were potentially infectious [17]. Energy devices also generate aerosols, which might be infective as well [18]. Adopting appropriate strategies to minimize infectious risks in addition to ensuring sufficient information sharing is essential for performing surgery for COVID-19 patients safely.

## Conclusions

We reported a case of colon perforation caused by COVID-19-induced coagulopathy. Although intestinal perforation is not common as a complication of COVID-19 pneumonia, it can be a differential diagnosis when a patient develops secondary sepsis.

## Data Availability

None.

## Abbreviations

APTT: Activated partial thromboplastin time
BP: Blood pressure
BT: Body temperature
COVID-19: Coronavirus disease
CRP: C-reactive protein
CT: Computed tomography
ECMO: Extracorporeal membrane oxygenation
HR: Heart rate
ICU: Intensive-care unit
PaCO_2_: Partial pressure of carbon dioxide
PaO_2_: Partial pressure of oxygen
PCR: Polymerase chain reaction
PEEP: Positive end-expiratory pressure
PiP: Peak inspiratory pressure
PT-INR: Prothrombin time-international normalized ratio
WBC: White blood cell counts
